# Protective effects of human umbilical cord mesenchymal stem cells-derived small extracelluar vesicles on corneal epithelial cells under hyperosmotic stress: Inhibition of oxidative damage and inflammation

**DOI:** 10.1590/1678-4685-GMB-2025-0026

**Published:** 2026-06-12

**Authors:** Chengyu Peng, Mingqi Zhang, Yuqing Bao, Zhuoshi Wang, Fenglei Zhang, Wei He

**Affiliations:** 1 Jinzhou Medical University, The Third Clinical Medical School, Jinzhou, Liaoning, China.; 2 He Eye Specialist Hospital, Shenyang, Liaoning, China.; 3 He University, Precision Medicine Innovation Institute, Stem Cell Center, Shenyang, Liaoning, China.; 4 He University, Liaoning Key Laboratory of Ophthalmic Stem Cells, Shenyang, Liaoning, China.

**Keywords:** Mesenchymal stem cells, small extracellular vesicles, hyperosmotic stress, oxidative damage, inflammation

## Abstract

Dry eye disease (DED) is often associated with corneal epithelial injury under hyperosmotic stress, which contributes to epithelial cell apoptosis and delay wound healing. Human Umbilical Cord Mesenchymal Stem Cell-derived Small Extracellular Vesicles (hUC-MSCs-sEVs) have emerged as promising therapeutic agents due to their anti-inflammatory, anti-apoptotic, and regenerative properties. In this study, we isolated and characterized hUC-MSCs-sEVs and assessed their therapeutic potential in hyperosmotic corneal epithelial injury. We demonstrated that hUC-MSCs-sEVs significantly promoted human corneal epithelial cell proliferation and repair under hyperosmotic conditions, reducing oxidative stress and preserving mitochondrial function. Additionally, hUC-MSCs-sEVs inhibited the expression of pro-inflammatory cytokines IL-6 and IL-1β, as well as downregulated the cGAS-STING signaling pathway, a critical mediator of inflammation. These findings suggest that hUC-MSCs-sEVs may offer a novel therapeutic strategy for treating hyperosmotic stress-induced corneal epithelial damage by mitigating oxidative stress, preserving mitochondrial integrity, and modulating inflammatory responses.

## Introduction

Dry eye disease (DED) is a prevalent ocular surface disorder characterized by tear film instability, inflammation, and corneal epithelial damage, ultimately leading to visual impairment and discomfort (Mohamed et al., 2022; Sheppard et al., 2023). Despite advances in treatment, current therapies remain limited in effectively restoring corneal epithelial integrity and reducing inflammation (Wong et al., 2023) Recently, mesenchymal stem cells (MSCs) have emerged as a promising therapeutic approach due to their potent regenerative and immunomodulatory properties (Song et al., 2020; Matsuzaka and Yashiro, 2024). Increasing evidence suggests that the therapeutic effects of MSCs are largely mediated through their secreted small extracellular vesicles (sEVs), which act as paracrine effectors facilitating intercellular communication (Marote et al., 2016). According to the MISEV2023 guidelines, sEVs represent a heterogeneous population of membrane-bound nanovesicles containing bioactive cargos such as proteins, lipids, and RNAs that modulate cellular processes including proliferation, migration, apoptosis, and immune signaling (Zhou et al., 2023; Welsh et al., 2024). Liu et al. (2022a)  demonstrated that human umbilical cord MSCs derived Small Extracellular Vesicles (hUC-MSCs-sEVs) could enhance corneal epithelial regeneration in a dry eye model. Tian et al. (2023)  reported that MSCs-sEVs could suppress oxidative stress-induced apoptosis in corneal epithelial cells. Moreover, hUC-MSCs-sEVs have been found to modulate immune responses and reduce inflammatory cytokine expression in ocular surface diseases (Lee et al., 2015). However, the role of hUC-MSCs-sEVs in hyperosmotic stress-induced corneal epithelial injury remains unclear.

The cyclic GMP-AMP synthase-stimulator of interferon genes (cGAS-STING) signaling pathway plays a crucial role in inflammation and cellular stress responses (Ding et al., 2020; Liu et al., 2022b). Activation of this pathway contributes to epithelial apoptosis and inflammatory cytokine production in dry eye (Yan et al., 2024a). Zhao *et al*. found that inhibiting cGAS-STING signaling alleviated corneal inflammation in experimental DED (Ouyang et al., 2023). However, whether hUC-MSCs-sEVs exert their protective effects via the cGAS-STING pathway remains unknown.

In the present study, we investigated the protective effects of hUC-MSCs-sEVs on hyperosmotic stress-induced corneal epithelial cell damage. We explored their anti-inflammatory and anti-oxidative mechanisms, with a focus on the potential involvement of the cGAS-STING signaling pathway. This research may provide a foundation for the clinical application of hUC-MSCs-sEVs in DED treatment.

## Material and Methods

### Identification of hUC-MSCs

Human umbilical cord mesenchymal stem cells (hUC-MSCs) were purchased from Shenyang Yuanchu Biotech (Shenyang, China) and authenticated by the supplier using standard STR profiling. The STR profiling results confirmed that hUC-MSCs matched the human cell origin database and had no cross-contamination with other cell lines, further verifying the identity and quality of the MSCs used in this study (Figure S1). Cells were routinely tested for mycoplasma contamination and confirmed to be mycoplasma-free before experiments. hUC-MSCs were cultured in α-MEM supplemented with 5% human platelet lysates (UltraGRO, Helios, EU) at 37 °C in a humidified atmosphere with 5% CO₂. The medium was refreshed every 2 days. MSC morphology was monitored under an inverted phase-contrast microscope (Olympus, Japan). Flow cytometry (NovoCyte, Agilent, USA) was used to analyze the expression of MSC surface markers CD90, CD105, and CD34. The multipotent differentiation potential of MSCs was assessed by inducing adipogenic, osteogenic, and chondrogenic differentiation, followed by staining with oil red O, alizarin red, and alcian blue, respectively (Cygan, China).

### Isolation and characterization of smal extracellular vesicles from hUC-MSCs (hUC-MSCs-sEVs)

sEVs were isolated from conditioned medium following the MISEV2023 guidelines (Théry et al., 2006). Briefly, hUC-MSCs were cultured in exosome-depleted FBS (RayBio, China) for 48 h.The supernatant was sequentially centrifuged at 300×g for 10 min, 3,000×g for 15 min, and 10,000×g for 20 min. The resulting supernatant was ultracentrifuged at 100,000×g for 2 h at 4 °C (Hitachi, CP80NX, Japan). The pellet was washed with phosphate-buffered saline (PBS) and centrifuged again at 80,000 ×g for 1 h, resuspended in 250 μL PBS and stored at -80 °C until further use. TEM (Hitachi HT7700) and nanoparticle tracking analysis (ZetaView, PMX-120) were used for morphology and size distribution. Western blotting confirmed CD9, CD63, and TSG101, and absence of calnexin.

### Labeling and uptake of sEVs

To track the internalization of sEVs by recipient cells, sEVs were labeled with PKH67 Green Fluorescent Cell Linker Kit (Sigma-Aldrich, USA) according to the manufacturer’s protocol. Briefly, 2 μL PKH67 solution was added to 100 μg sEVs and incubated for 15 minutes at room temperature. Then the mixture was added to 18 mL PBS and centrifuged at 120,000×g for 2 hours at 4 °C. The supernatant was removed, and the pellet was resuspended in 0.2 mL PBS and centrifuged at 120,000×g for another 2 hours at 4 °C. The PKH67-labeled sEVs were resuspended in 200 μL PBS and then added to the cells. After incubation for 5 h, Nuclei were counterstained with DAPI to visualize intracellular localization by fluorescence microscopy (Olympus, Japan).

### Hyperosmotic stress model and corneal epithelial scratch assay

Human corneal epithelial cells (HCECs, obtained from Guangzhou, China) were cultured in DMEM/F12 medium supplemented with 10% FBS, 100 U/mL penicillin, and 100 μg/mL streptomycin in a humidified 5% CO₂ atmosphere at 37 °C. To induce hyperosmotic stress, cells were incubated in a hyperosmolar medium (110 mOsm/kg) prepared by adding NaCl (Sinopharm Chemical Reagent Co., Ltd. Cat10019318, China) to the culture medium for 24 h. For the corneal epithelial scratch assay, a linear wound was created using a 200-μL pipette tip. Cells were washed with 0.1% HAS and treated with hUC-MSCs-sEVs (4 μg/mL) for 24, 48, and 72 h. The wound healing process was observed under an inverted microscope, and the wound closure rate was analyzed using ImageJ software. A transwell migration assay was performed to evaluate sEV-mediated migration enhancement. Cell migration was evaluated using 24-well Transwell inserts with 8-μm pore membranes (Corning, USA). The lower chamber was filled with 600 μL of culture medium containing 10% fetal bovine serum (FBS) as a chemoattractant. The upper chamber was seeded with 5 × 10⁴ human corneal epithelial cells (HCECs) suspended in 100 μL of medium and treated as indicated. After incubation at 37 °C with 5% CO₂ for 48 h, cells on the upper membrane surface were gently removed with a cotton swab. The migrated cells on the lower surface were fixed and stained with Giemsa solution, rinsed with PBS, and imaged under a microscope. Three random fields per insert were photographed, and migrated cells were quantified using ImageJ software. 

### Reactive oxygen species (ROS) assay

Intracellular ROS levels were measured using the DCFH-DA probe (Beyotime, China). Cells were incubated with 10 μM DCFH-DA at 37 °C for 30 min in the dark, followed by washing with PBS. Fluorescence intensity was analyzed by Image J software.

### Mitochondrial membrane potential (ΔΨm) assay

The mitochondrial membrane potential was assessed using the JC-1 dye (Beyotime, China). Cells were stained with 10 μM JC-1 dye at 37 °C for 30 min and washed with PBS. The fluorescence intensity was analyzed by Image J software. The ΔΨm ratio was calculated as the red/green fluorescence intensity.

### Western blot and ELISA

The cells from each group were collected and lysed with RIPA lysis buffer (invitrogen, Gibco, USA), and the protein concentrations in each group were determined by BCA method. (Beyotime, China). Lysates in equal amounts of 20 μg proteins were separated by SDS-PAGE (Bio-Rad, USA) and then transferred to PVDF membranes (Pall Corporation, USA). After rinsing with TBS (Beyotime, China) several times and blocking with 5% non-fat milk (BBI, China), the membranes were incubated with anti-cGAS primary antibody (26416-1-AP, Proteintech, China), anti-sting primary antibody (19851-1-AP, Proteintech,China), anti-IL-6 (218651-1-AP, Proteintech,China) and anti-IL-1β (16806-1-AP, Proteintech,China) overnight. Followed by thoroughly washing, HRP conjugated secondary antibodies (SA00001-2, Proteintech, China) were incubated with membranes in darkness for 1 h. ECL reagent (Tanon, China) was added to the membranes to visualize the immunoreactive protein bands, and the ChemiDoc MP imaging system (Bio Rad, USA) was used to analyze. These supernatants were used to evaluate the secretion of inflammatory cytokines (IL-1β and IL-6) via ELISA assay following the manufacturer’s protocol (Wuhan Huawei Biotech, China). 

### Statistical analysis

All experiments were performed at least three times. Data were analyzed using GraphPad Prism 9.0 software and presented as mean±standard deviation (SD). Differences between groups were compared using one-way ANOVA followed by Bonferroni post hoc test. A p-value < 0.05 was considered statistically significant.

## Results

### Characterization of hUC-MSCs and their derived sEVs

**Figure 1-:**
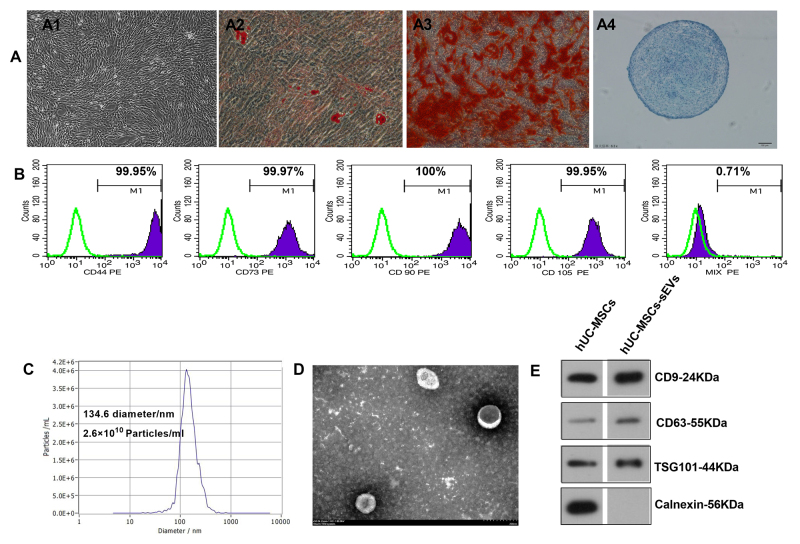
Characterization of mesenchymal stem cells derived from human umbilical cord (hUC-MSCs) and their derived small extracellular vesicles (hUC-MSCs-sEVs). (A) Morphology and trilineage differentiation of hUC-MSCs. Scale bar = 200 μm. A2-adipocytes differentiation (formation of lipid droplets stained with Oil Red); A3-osteocytes differentiation (demonstrated by calcium deposition stained with Alizarin Red) and A4-chondrocytes differentiation (demonstrated by deposition of extracellular matrix stained with alcian blue. (B) Surface marker profiling was determined by flow cytometry. (C) Nanoparticle Tracking Analysis (NTA) of isolated **hUC-MSCs-sEVs**. (D) Transmission Electron Microscopy (TEM) Images of hUC-MSCs-Exo. TEM images depicting the morphology of **hUC-MSCs-sEVs** at 20000 nm scale. The vesicles appear as spherical particles, indicating successful isolation and preservation of their structural integrity. (E) Western blot analysis of hUC-MSCs and their derived sEVssurface marker.

hUC-MSCs exhibited a fibroblast-like morphology. Adipogenic differentiation was confirmed by oil red O staining, showing lipid droplet accumulation. Osteogenic differentiation was validated by alizarin red staining, indicating calcified nodule formation (Figure 1A). Additionally, alcian blue staining confirmed chondrogenic differentiation with cartilage-like spheroid formation. Flow cytometry analysis showed that hUC-MSCs expressed CD44, CD73, CD90 and CD105 but lacked CD34, CD45, CD11b, CD19, and HLA-DR (Figure 1B). TEM revealed hUC-MSCs-Small Extracellular Vesicles (hUC-MSCs-sEVs) with a double-membrane, disc-like structure, and NTA showed an average size of 134.6 nm. The detailed NTA report is presented in Figure S2. Western blot confirmed the presence of CD9, CD63, and TSG101 in hUC-MSCs-sEVs, while calnexin was absent. In contrast, hUC-MSCs expressed all markers, including calnexin (Figure 1). The analysis of these protein bands is shown in Figure S3. These results confirm the successful isolation and characterization of hUC-MSC-derived sEVs.

### hUC-MSCs-sEVs protect human corneal epithelial cells under hypertonic stress

**Figure 2 -:**
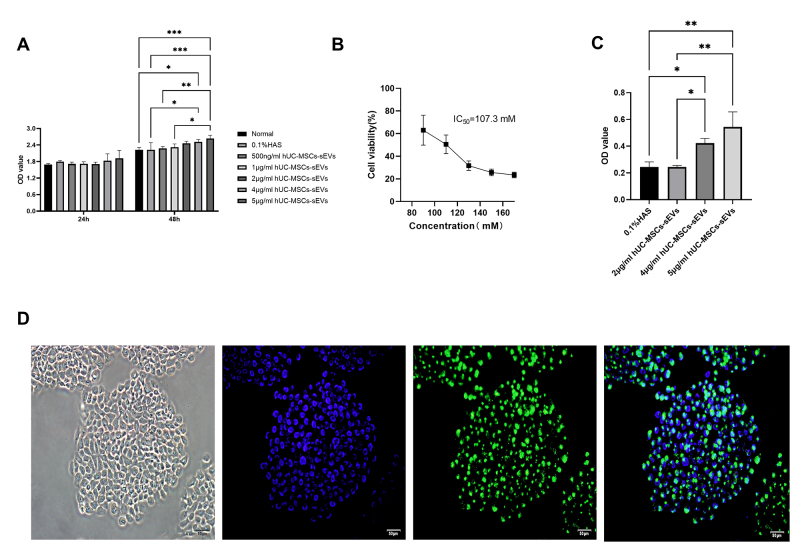
The effect of hUC-MSCs-sEVs on human corneal epithelial cells (A) Impact of various concentrations of hUC-MSCs-sEVs on human corneal epithelial cells proliferation. (B) Calculation of IC50 concentration for NaCl on human corneal epithelial cells proliferation. (C) HCECs were first exposed to 110 mM NaCl for 24 h to induce hyperosmotic injury, followed by treatment with hUC-MSC-sEVs for an additional 48 h. Cell viability was then assessed using the CCK-8 assay. (D) Representative images of the epithelial cells uptake of PHK67 (Green) labelled hUC-MSCs-Exo, scale bar: 50 μm, *p < 0.05, **p < 0.01, ***p < 0.001.

An *in vitro* model of hypertonic stress was established in normal human corneal epithelial cells (hCECs). The effect of different concentrations of hUC-MSCs-sEVs on corneal epithelial cell proliferation was tested, with 0.1% hyaluronate sodium (0.1%HSA) as the control group. As shown in Figure 2A, after 48 hours, hUC-MSCs-sEVs at a concentration of 4 µg/ml significantly promoted the proliferation of hCECs (P < 0.05), and the effect increased with the concentration of hUC-MSCs-sEVs (P<0.001, at the concentration of 5µg/ml hUC-MSCs-sEVsvs. Normal cell group and 0.1% HAS group) Subsequently, the IC50 value of NaCl was determined to be 107.3 mM, and 110 mM NaCl was chosen for later experiments (Figure 2B). HCECs were first exposed to 110 mM NaCl for 24 hours to induce hyperosmotic injury, followed by treatment with hUC-MSC-sEVs for an additional 48 hours. As shown in Figure 2C, hUC-MSC-sEVs exhibited significant protective effects on HCECs under these conditions. No significant difference was observed between the 4 µg and 5 µg hUC-MSCs-sEVs concentrations. Therefore, 4 µg of hUC-MSCs-sEVs was selected for further analysis. Additionally, Figure 2D shows the phagocytosis of hUC-MSCs-sEVs by hCECs after 5 hours co-culture, confirming the uptake of sEVs by the cells under hypertonic conditions.

### hUC-MSCs-sEVs promote human corneal epithelial cell wound healing

**Figure 3-:**
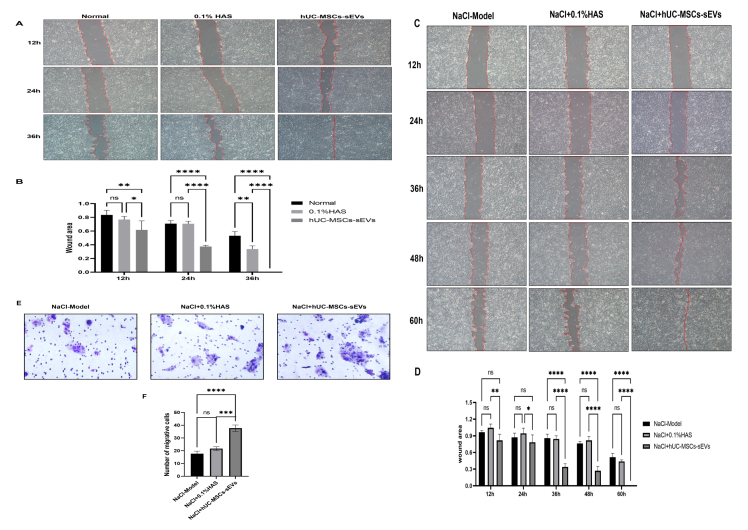
hUC-MSCs-sEVs effectively promote human corneal epithelial cells wound healing (A) Microscopic images of human corneal epithelial cell damage repair at different time points at normal damage, scale bar= 200 μm. (B) Comparative analysis of human corneal epithelial cell damage area at different time points. (C) Microscopic images of human corneal epithelial cell damage repair at different time points under hypertonic stress, scale bar= 200 μm. (D) Comparative analysis of human corneal epithelial cell damage area at different time points. € Representative images of migrated HCECs in Transwell assays under hyperosmotic conditions. Cells that traversed the membrane were stained with crystal violet (purple). Scale bar = 100 μm. (F) Quantification of migrated cells, ****p < 0.0001, ***p < 0.001, **p < 0.01, *p <0.05, ns means not significant.

In a normal scratch assay, 4 µg/ml of hUC-MSCs-sEVs treatment significantly accelerated wound healing at 12 hours (P<0.5, vs 0.1% HAS, P<0.01, vs normal control) and complete wound closure was observed at 36 hours in hUC-MSCs-sEVs group (Figure 3A, B). Subsequently, in the NaCl-induced hypertonic model, after 36 hours, hUC-MSCs-sEVs treatment significantly enhanced the proliferation of corneal epithelial cells compared to both the model and 0.1% HAS groups (P<0.0001). By 60 hours, the hUC-MSCs-sEVs -treated group exhibited complete wound healing, demonstrating the sEVs’ potential in promoting the repair of corneal epithelial damage under both normal and hypertonic stress conditions (Figure 3C, D).

To further validate whether hUC-MSC-sEVs promote wound repair primarily by enhancing epithelial cell migration rather than proliferation, a Transwell migration assay was performed. HCECs treated with hUC-MSC-sEVs (4 μg/mL) exhibited a significantly higher number of migrated cells compared with 0.1% HAS and hyperosmotic model groups (P< 0.001, vs 0.1% HAS, P< 0.0001, vs NaCl Model). This result confirms that hUC-MSC-sEVs promote corneal epithelial repair through enhancing cell migratory capacity in addition to their proliferative effects. The representative images and quantitative analysis are presented in Figure 3E and 3F.

### hUC-MSCs-sEVs protect human corneal epithelial cell from oxidative damage under hypertonic stress

**Figure 4-:**
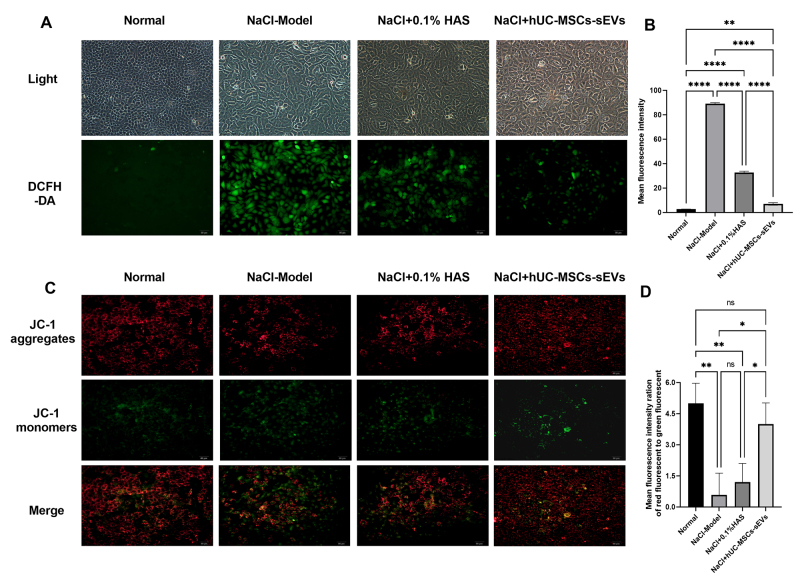
hUC-MSCs-sEVs alleviate hypertonic induced oxidative stress and oxidative damage in human corneal epithelial cells (A) Cells were stained with DCFH-DA, and intracellular ROS levels were observed by a fluorescent microscopy. Scar bar=50 μm. (B)Statistics of intracellular ROS level. (C) Fluorescence imaging of HCECs stained with JC-1 in different treatment groups. (D) The aggregate/monomer fluorescence intensity ration of JC-1 in HCECs. ****p < 0.0001, ***p < 0.001, **p < 0.01, *p <0.05, ns means not significant.

hUC-MSCs-sEVs treatment effectively inhibited the production of reactive oxygen species (ROS) in corneal epithelial cells exposed to hypertonic stress (Figure 4A, B). Mitochondrial membrane potential was assessed using JC-1 staining, which revealed that hUC-MSCs-sEVs treatment significantly protected the mitochondrial membrane potential in corneal epithelial cells (Figure 4C). The red-to-green fluorescence ratio indicated that sEVs significantly protected corneal epithelial cells from hypertonic-induced oxidative damage, preserving mitochondrial integrity (Figure 4D). These results suggest that hUC-MSCs-sEVs can play a crucial role in mitigating oxidative stress and protecting cellular function under high osmotic conditions.

### hUC-MSCs-sEVs inhibit cGAS-STING signaling pathway and inflammatory cytokine expression

**Figure 5-:**
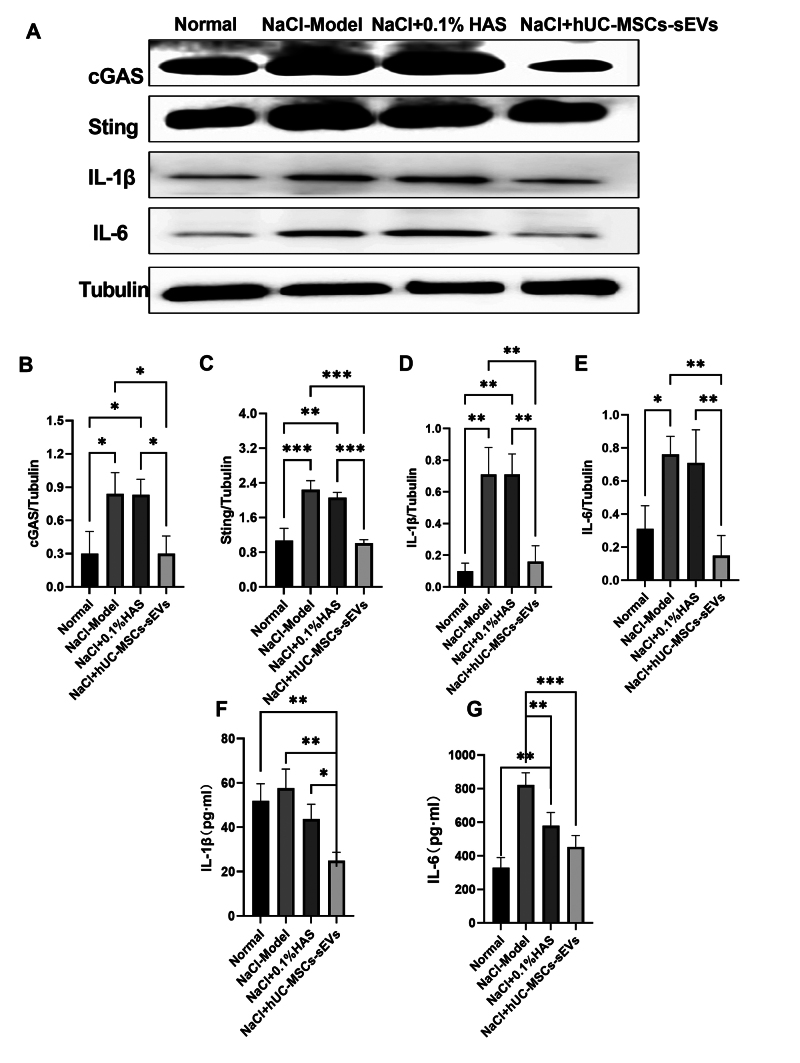
cCAS-STING signaling pathway is involved in the effect of hUC-MSCs-sEVs on human corneal epithelial cells A-E Expression levels of cGAS, Sting, IL-1β, and IL-6 in hypertonic induced HCECs were detected by western blotting. (F&G) ELISA analysis of IL-1β, and IL-6 secretion by HCECs under hyperosmotic conditions after treatment with hUC-MSCs-sEVs. ***p < 0.001, **p < 0.01, *p <0.05.

The cGAS-STING signaling pathway plays a critical role in the regulation of immune responses and inflammation. In this study, the expression of cGAS and STING was significantly increased in the model group compared to the NC group (P < 0.001). However, hUC-MSCs-sEVs treatment significantly decreased the expression of cGAS, STING, and the inflammatory cytokines IL-6 and IL-1β in the sEVs-treated group. The detailed western blotting results of cGAS, STING, IL-1β and IL-6 expression are provided in Figure S4. Compared with the model group, the expression levels of these markers were significantly lower in the sEVs group (P < 0.001), suggesting that hUC-MSCs-sEVs effectively inhibit the cGAS-STING signaling pathway and reduce inflammatory responses (Figure 5A-E). ELISA results demonstrated the downregulation of IL-6 and IL-1β in the sEVs-treated group (Figure 5F, G).

## Discussion

Corneal epithelial injury under hyperosmotic stress is a key pathological feature of dry eye disease (DED) (Yang et al., 2019). Previous studies have demonstrated that mesenchymal stem cell-derived small extracellular vesicles (hUC-MSCs-sEVs) exert protective effects in various disease models through their anti-inflammatory (Nakano and Fujimiya, 2021), anti-apoptotic (Xia et al., 2021), and regenerative properties (Rayat Pisheh and Sani, 2021). In this study, we successfully isolated and characterized hUC-MSCs-sEVs, confirmed the expression of classical EV markers (CD9, CD63, and TSG101), and demonstrated their therapeutic potential in hyperosmotic corneal epithelial injury.

Our results demonstrated that hUC-MSCs-sEVs significantly promoted corneal epithelial cell repair under hyperosmotic conditions. This protective effect was associated with the attenuation of oxidative damage, the preservation of mitochondrial membrane function. Excessive oxidative stress and mitochondrial dysfunction are critical factors contributing to epithelial cell apoptosis, impaired migration, and delayed wound healing in DED (Ouyang et al., 2024). Importantly, sEV treatment reduced intracellular ROS accumulation and stabilized mitochondrial function, indicating that their cytoprotective effect involves both antioxidant and mitochondrial-preserving mechanisms.

Beyond wound repair, oxidative stress in corneal epithelial cells also influences several signaling pathways associated with immune activation, metabolic adaptation, and epithelial barrier maintenance (Böhm et al., 2023). By mitigating oxidative injury, hUC-MSC-sEVs may indirectly regulate redox-sensitive transcription factors such as NF-κB and Nrf2, thereby promoting a more balanced cellular response and supporting homeostasis under hyperosmotic challenge (Che et al., 2024; Shahrezaei et al., 2025).

Inflammation plays a crucial role in the progression of hyperosmotic stress-induced corneal epithelial injury (Yan et al., 2024b). In our study, we observed a significant downregulation of pro-inflammatory cytokines IL-6 and IL-1β following sEVs treatment. Notably, we found that the expression of stimulator of interferon genes (STING), a key component of the cGAS-STING signaling pathway, was also significantly decreased. The cGAS-STING pathway is known to be involved in innate immune activation and chronic inflammation, which are critical in the pathogenesis of DED (Tan et al., 2024). Inhibition of this pathway by sEVs suggests that their anti-inflammatory action may involve limiting cytosolic DNA release and mitochondrial stress signaling, thereby preventing downstream activation of inflammatory cytokines.

Furthermore, excessive STING activation has been linked to mitochondrial dysfunction, reactive oxygen species generation, and apoptotic cell death (Yan et al., 2022). The observed suppression of STING in the hUC-MSC-sEVs -treated groupindicates that these vesicles protect corneal epithelial cells not only by attenuating inflammation but also by preserving mitochondrial integrity. This observation aligns with previous studies showing that inhibition of the cGAS-STING pathway can alleviate inflammation and oxidative stress in various disease models (Huang et al., 2022; Zhang et al., 2024).

The molecular cargo carried by sEVs may underlie these beneficial effects. Previous studies have identified regulatory microRNAs such as miR-21, miR-146a, and miR-181a, as well as antioxidant enzymes and anti-apoptotic proteins within MSC-derived sEVs. These molecules are known to modulate pathways related to oxidative stress, inflammation, and cellular migration (Zheng et al., 2024; Zhou et al., 2025). It is plausible that the observed reduction in ROS and cGAS-STING activation in our study is mediated by these functional cargos. In addition, our transwell migration assays indicate that sEVs enhance epithelial migration, suggesting that their wound-healing effects may involve both proliferative and motility-enhancing mechanisms.

In conclusion, our study provides evidence that hUC-MSCs-sEVs significantly promote corneal epithelial repair under hyperosmotic stress by reducing oxidative damage preserving mitochondrial function, and suppressing cGAS-STING-mediated inflammation. By maintaining mitochondrial homeostasis and modulating innate immune signaling, hUC-MSC-sEVs offer a promising cell-free therapeutic strategy for treating hyperosmotic stress-induced corneal epithelial injury and potentially other oxidative stress-related ocular surface disorders. Future *in vivo* studies and detailed molecular analyses of sEV cargo composition are warranted to further elucidate their mechanism of action and optimize their clinical translation for dry eye disease therapy.

## Data Availability

The authors confirm that the data supporting the findings of this study are available within the article.

## Supplementary material

Figure S1 - Short tandem repeat (STR) profiling of human umbilical cord mesenchymal stem cells (hUC-MSCs).

Figure S2 - Nanoparticle tracking analysis (NTA) report of hUC-MSCs-derived sEVs (detected by ZetaView PMX-120, Particle Metrix, Germany).

Figure S3 - Representative original Western blot images of small extracellular vesicles (sEVs) isolated from hUC-MSCs.

Figure S4 - Unprocessed images of the Western blots shown in Figure 5.
