# Supplementary material for: Protective effects of human umbilical cord mesenchymal stem cells-derived small extracelluar vesicles on corneal epithelial cells under hyperosmotic stress: Inhibition of oxidative damage and inflammation
Source: Genet Mol Biol. 2026 Jun 12;49(2):e20250026. doi: 10.1590/1678-4685-GMB-2025-0026 (PMC13262691; doi:10.1590/1678-4685-GMB-2025-0026)
Supplement: Figure S1 [file 1415-4757-GMB-49-2-e20250026-s1.pdf]

Genetics and Molecular Biology
https://doi.org/10.1590/1678-4685-GMB-2025-0026

Supplementary Material to “Protective effects of human umbilical cord mesenchymal stem cells-derived small extracelluar vesicles on corneal epithelial cells under hyperosmotic stress: Inhibition of oxidative damage and inflammation”

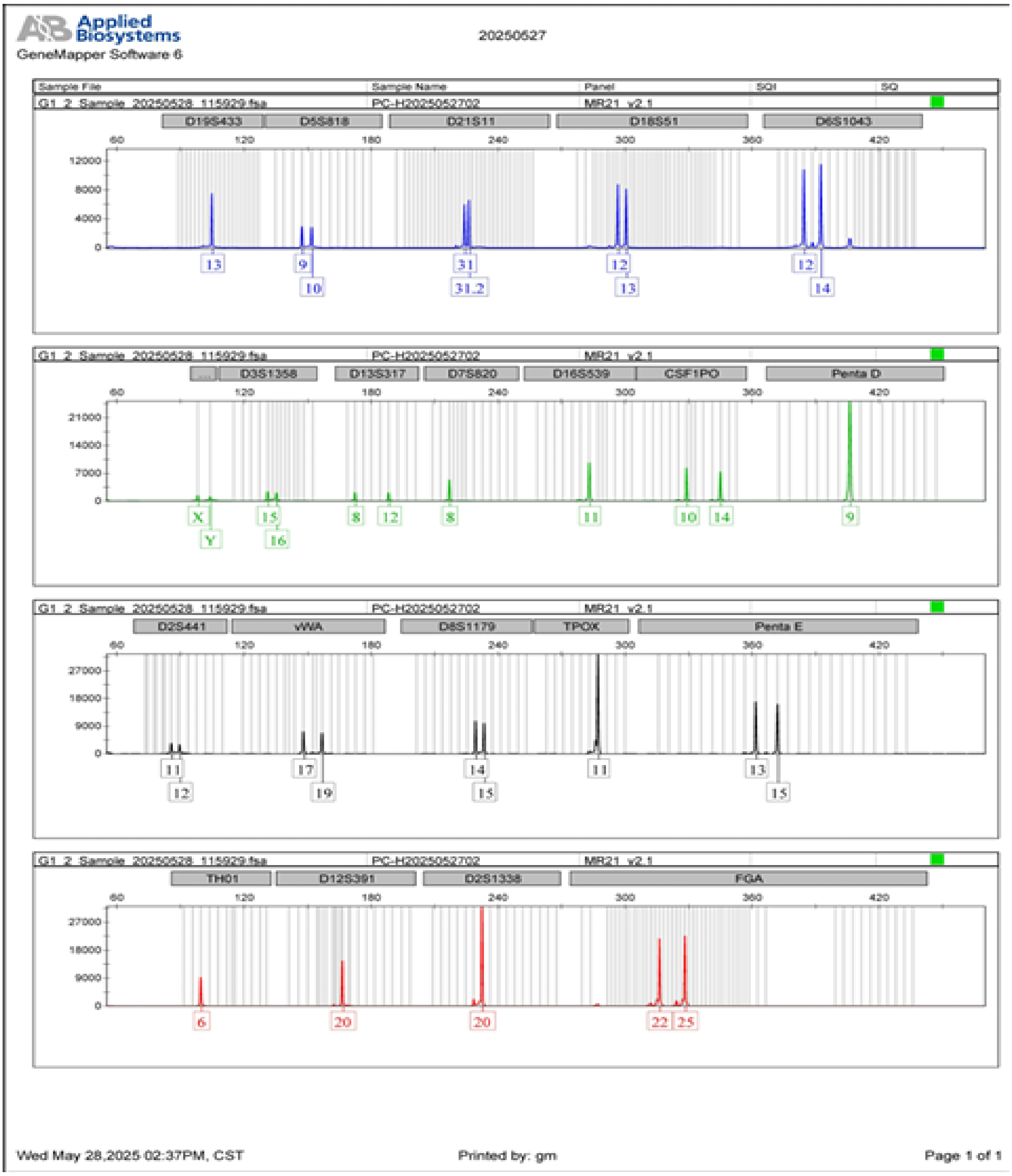

Figure S1 - Short tandem repeat (STR) profiling of human umbilical cord mesenchymal stem cells(hUC-MSCs). The STR analysis confirmed the identity and human origin of the MSCs used in this study, ensuring the authenticity and quality of the cell line.
