# Supplementary material for: Protective effects of human umbilical cord mesenchymal stem cells-derived small extracelluar vesicles on corneal epithelial cells under hyperosmotic stress: Inhibition of oxidative damage and inflammation
Source: Genet Mol Biol. 2026 Jun 12;49(2):e20250026. doi: 10.1590/1678-4685-GMB-2025-0026 (PMC13262691; doi:10.1590/1678-4685-GMB-2025-0026)
Supplement: Figure S2 [file 1415-4757-GMB-49-2-e20250026-s2.pdf]

Genetics and Molecular Biology
https://doi.org/10.1590/1678-4685-GMB-2025-0026

Supplementary Material to "Protective effects of human umbilical cord mesenchymal stem cells-derived small extracelluar vesicles on corneal epithelial cells under hyperosmotic stress: Inhibition of oxidative damage and inflammation"

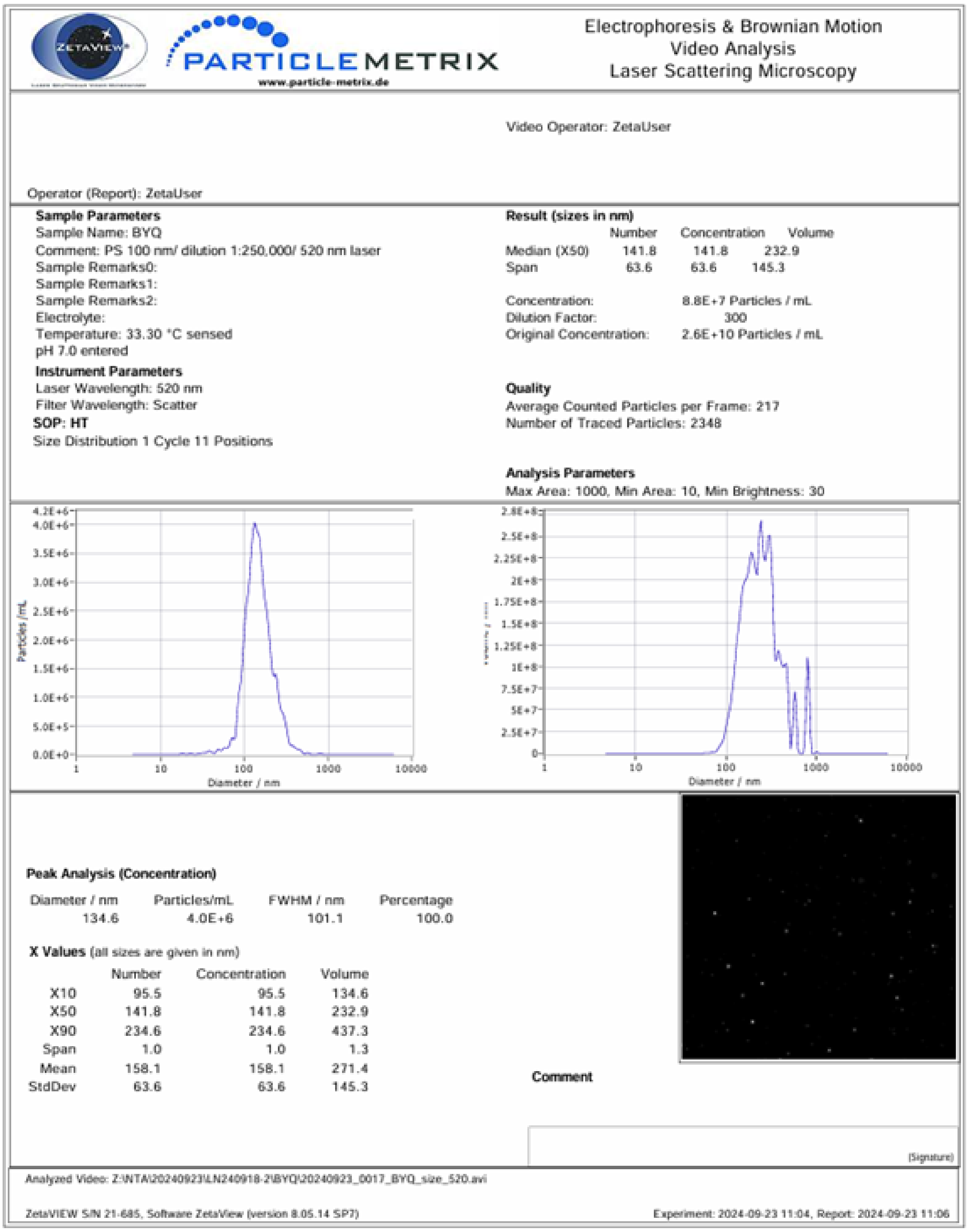

PARTICLEMETRIX
www.particle-metrix.de

Electrophoresis & Brownian Motion
Video Analysis
Laser Scattering Microscopy

Video Operator: ZetaUser

Operator (Report): ZetaUser

Sample Parameters
Sample Name: BYQ
Comment: PS 100 nm/ dilution 1:250,000/ 520 nm laser
Sample Remarks0:
Sample Remarks1:
Sample Remarks2:
Electrolyte:
Temperature: 33.30 °C sensed
pH 7.0 entered

Instrument Parameters
Laser Wavelength: 520 nm
Filter Wavelength: Scatter

SOP: HT
Size Distribution 1 Cycle 11 Positions

Result (sizes in nm)

	Number	Concentration	Volume
Median (X50)	141.8	141.8	232.9
Span	63.6	63.6	145.3

Concentration: 8.8E+7 Particles / mL
Dilution Factor: 300
Original Concentration: 2.6E+10 Particles / mL

Quality
Average Counted Particles per Frame: 217
Number of Traced Particles: 2348

Analysis Parameters
Max Area: 1000, Min Area: 10, Min Brightness: 30

Peak Analysis (Concentration)

Diameter / nm	Particles/mL	FWHM / nm	Percentage
134.6	4.0E+6	101.1	100.0

X Values (all sizes are given in nm)

	Number	Concentration	Volume
X10	95.5	95.5	134.6
X50	141.8	141.8	232.9
X90	234.6	234.6	437.3
Span	1.0	1.0	1.3
Mean	158.1	158.1	271.4
StdDev	63.6	63.6	145.3

Comment

(Signature)

Analyzed Video: Z:\NTA\20240923\LN240918-2\BYQ\20240923_0017_BYQ_size_520.avi

ZetaVIEW S/N 21-685, Software ZetaView (version 8.05.14 SP7)

Experiment: 2024-09-23 11:04, Report: 2024-09-23 11:06

Figure S2 - Nanoparticle tracking analysis (NTA) report of hUC-MSCs-derived sEVs. (detected by ZetaView PMX-120, Particle Metrix, Germany). Particle size distribution histogram (main peak: 134.6 nm); Statistical summary of particle concentration (2.6×10^{10} particles/mL).
