# Supplementary material for: Protective effects of human umbilical cord mesenchymal stem cells-derived small extracelluar vesicles on corneal epithelial cells under hyperosmotic stress: Inhibition of oxidative damage and inflammation
Source: Genet Mol Biol. 2026 Jun 12;49(2):e20250026. doi: 10.1590/1678-4685-GMB-2025-0026 (PMC13262691; doi:10.1590/1678-4685-GMB-2025-0026)
Supplement: Figure S3 [file 1415-4757-GMB-49-2-e20250026-s3.pdf]

Genetics and Molecular Biology
https://doi.org/10.1590/1678-4685-GMB-2025-0026

Supplementary Material to “Protective effects of human umbilical cord mesenchymal stem cells-derived small extracelluar vesicles on corneal epithelial cells under hyperosmotic stress: Inhibition of oxidative damage and inflammation”

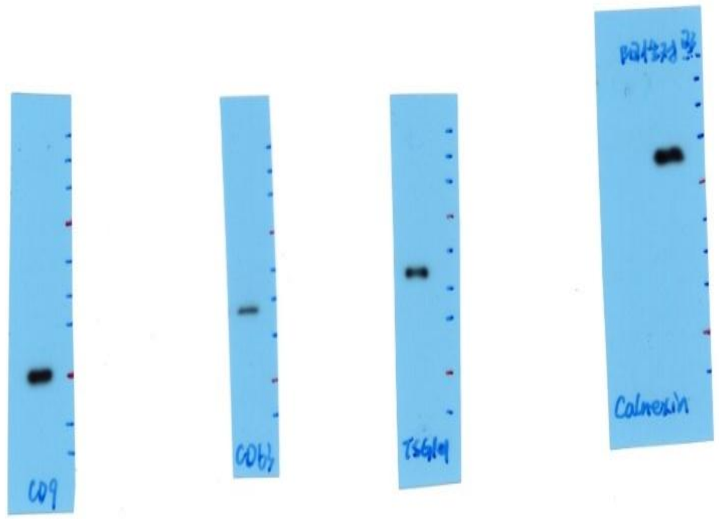

Figure S3 - Representative original Western blot images of small extracellular vesicles (sEVs) isolated from hUC-MSCs. The blots show the expression of sEV markers, including CD9, CD63, and TSG101. Full-length, uncropped images are presented to demonstrate the specificity and quality of the protein detection.
