# Supplementary material for: Protective effects of human umbilical cord mesenchymal stem cells-derived small extracelluar vesicles on corneal epithelial cells under hyperosmotic stress: Inhibition of oxidative damage and inflammation
Source: Genet Mol Biol. 2026 Jun 12;49(2):e20250026. doi: 10.1590/1678-4685-GMB-2025-0026 (PMC13262691; doi:10.1590/1678-4685-GMB-2025-0026)
Supplement: Figure S4 [file 1415-4757-GMB-49-2-e20250026-s4.pdf]

Genetics and Molecular Biology
https://doi.org/10.1590/1678-4685-GMB-2025-0026

Supplementary Material to "Protective effects of human umbilical cord mesenchymal stem cells-derived small extracelluar vesicles on corneal epithelial cells under hyperosmotic stress: Inhibition of oxidative damage and inflammation"

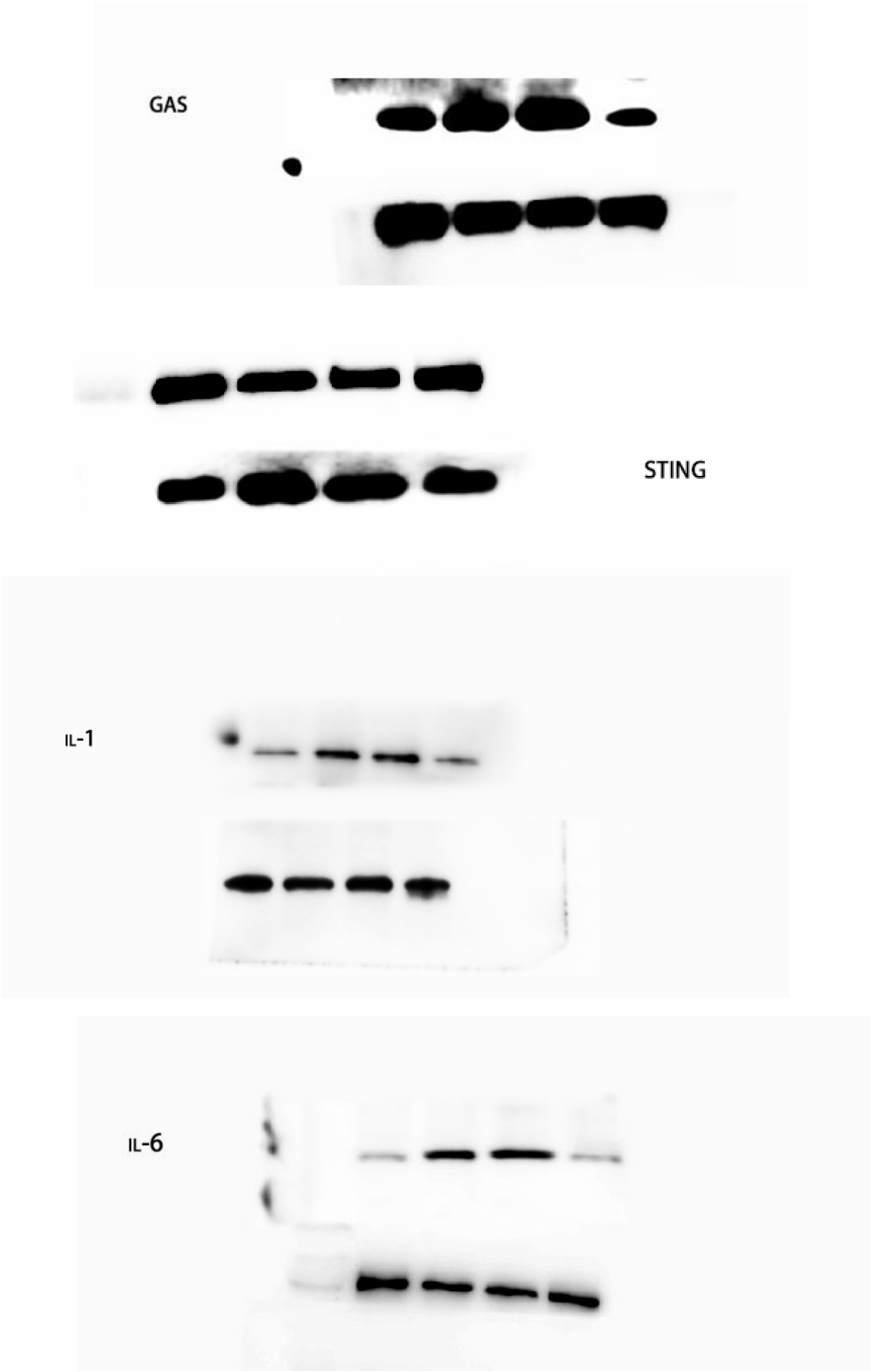

Figure S4 - Unprocessed images of the Western blots shown in Figure 5. These images are presented to demonstrate the specificity and integrity of protein detection.
